# 10-fs-level synchronization of photocathode laser with RF-oscillator for ultrafast electron and X-ray sources

**DOI:** 10.1038/srep39966

**Published:** 2017-01-09

**Authors:** Heewon Yang, Byungheon Han, Junho Shin, Dong Hou, Hayun Chung, In Hyung Baek, Young Uk Jeong, Jungwon Kim

**Affiliations:** 1 School of Mechanical and Aerospace Engineering, Korea Advanced Institute of Science and Technology (KAIST), Daejeon 34141, South Korea; 2 Center for Quantum-Beam-based Radiation Research, Korea Atomic Energy Research Institute (KAERI), Daejeon 34057, South Korea; 3 Department of Physics, Hannam University, Daejeon 34430, South Korea; 4 University of Electronic Science and Technology of China, Chengdu 610054, China; 5 Department of Electronics and Information Engineering, Korea University, Sejong 30019, South Korea

## Abstract

Ultrafast electron-based coherent radiation sources, such as free-electron lasers (FELs), ultrafast electron diffraction (UED) and Thomson-scattering sources, are becoming more important sources in today’s ultrafast science. Photocathode laser is an indispensable common subsystem in these sources that generates ultrafast electron pulses. To fully exploit the potentials of these sources, especially for pump-probe experiments, it is important to achieve high-precision synchronization between the photocathode laser and radio-frequency (RF) sources that manipulate electron pulses. So far, most of precision laser-RF synchronization has been achieved by using specially designed low-noise Er-fibre lasers at telecommunication wavelength. Here we show a modular method that achieves long-term (>1 day) stable 10-fs-level synchronization between a commercial 79.33-MHz Ti:sapphire laser oscillator and an S-band (2.856-GHz) RF oscillator. This is an important first step toward a photocathode laser-based femtosecond RF timing and synchronization system that is suitable for various small- to mid-scale ultrafast X-ray and electron sources.

Direct visualization of ultrafast phenomena with atomic spatial resolution and femtosecond temporal resolution is one of the key challenges pursued in today’s science. To achieve this goal, there have recently been intense research and development efforts for generating femtosecond X-ray pulses and electron pulses from FELs, UED and Thomson-scattering sources. The operation of these ultrafast X-ray/electron sources is based on well-coordinated interplay between ultrafast optical lasers and RF-driven electron accelerators. Ultrafast optical lasers, such as femtosecond Ti:sapphire mode-locked lasers, are used to generate electron pulses by photoelectric effect (as a photocathode laser) and to excite ultrafast phenomena in pump-probe experiments (as a pump laser). On the other hand, electron pulse shaping, manipulation, and acceleration are achieved by RF-based subsystems such as RF-photoguns and RF-driven accelerating cavities. Therefore, to fully exploit the potentials of these ultrafast electron/X-ray machines, especially for ultimate temporal resolutions in pump-probe experiments, precise synchronization between ultrafast optical lasers (solid-state mode-locked lasers in particular) and RF signals is first required.

In recent years, precise laser-RF synchronization with (sub-)femtosecond residual timing jitter and drift has been achieved by several different methods^1^,^2^. Most of previous demonstrations have been based on low-noise mode-locked Er-fibre lasers operating at telecommunication wavelength (~1550 nm). The use of low-noise mode-locked Er-fibre laser as the optical master oscillator (OMO) is an excellent way to achieve an effectively synchronized network for large-scale FEL facilities by distributing the OMO output to multiple remote locations by optical fibre links^1^,^3^. For small- to mid-scale FELs, UED or Thomson-scattering sources, however, operating a separate OMO with fibre distribution links for laser-RF synchronization is often costly and unnecessary. Since such facilities use a mode-locked solid-state (such as Nd:YAG^4^, Nd:YLF^5^, Yb:YAG^6^ and Ti:sapphire^7^,^8^,^9^,^10^,^11^,^12^,^13^,^14^,^15^) laser as both a photocathode laser for electron pulse generation and a pump laser for pump-probe experiments, direct synchronization of the mode-locked solid-state laser to RF oscillator is highly desirable. Even in large-scale facilities, achieving precise synchronization between mode-locked solid-state laser and RF oscillator is an important and useful technique as well, for example, for synchronization of seed lasers in seeded X-ray FELs^3^.

Among many different solid-state laser systems used for photocathode lasers, Ti:sapphire lasers are one of the most widely used laser systems^8^,^9^,^10^,^11^,^12^,^13^,^14^,^15^. There have been several prior works for tens-fs-precision synchronization between a Ti:sapphire laser oscillator and an RF oscillator in the past few years. Double-balanced microwave mixer-based synchronization methods have been extensively used^7^,^8^,^9^,^10^,^11^,^12^, which shows typical performance of ~80 fs residual jitter (10 Hz–100 kHz integration range) and ~90 fs drift over 15 minutes^9^. More recently, a much better result of <20 fs residual jitter (10 Hz–10 MHz integration range) was also demonstrated using mixer-based method^12^. Another recent work using a Mach-Zehnder modulator (MZM)-based RF synchronization addressed the long-term stability issue and showed an impressive performance of 4-fs long-term drift over 18 hours^13^,^14^. Note that the accurate short-term residual timing jitter spectrum was not measured in this work, and it is estimated that sub-20-fs-level synchronization is possible^15^.

Here, as a first step toward photocathode laser-based RF timing and synchronization for small- to mid-scale ultrafast X-ray and electron sources, we propose a modular synchronization method between a Ti:sapphire laser oscillator and an RF oscillator. The full time-domain and frequency-domain characterization results show 3.9-fs (rms) short-term residual timing jitter (integrated from 10 Hz to 100 kHz offset frequency) and 12.5-fs (rms) long-term residual timing drift over 24 hours, when synchronizing a commercial 79.33-MHz Ti:sapphire photocathode laser with an S-band (2.856-GHz in this work) RF oscillator.

## Results

### Characterization of timing jitter of a photocathode Ti:sapphire laser

**Figure 1:**
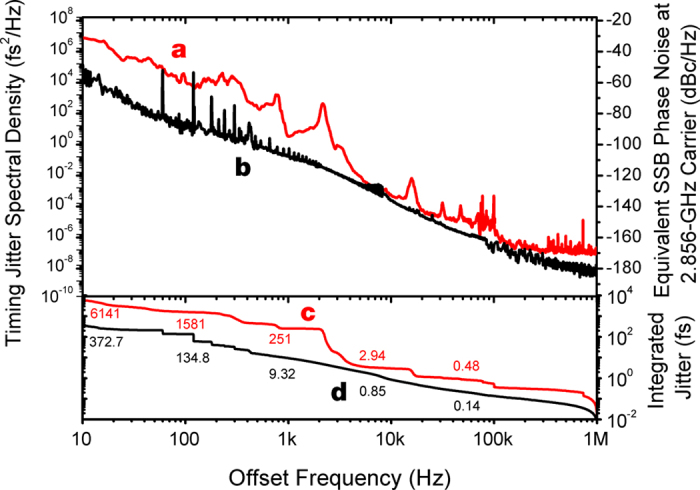
(**a**) Timing jitter PSD measurement result of the Ti:sapphire photocathode laser used in this work. (**b**) Timing jitter PSD measurement result of a typical stretched-pulse Er-fibre laser for comparison. (**c**) Integrated timing jitter (integration of curve **a**). (**d**) Integrated timing jitter (integration of curve **b**).

Despite low quantum-limited timing jitter enabled by short pulsewidth and high pulse energy^16^, mode-locked Ti:sapphire lasers suffer from large timing jitter in the lower offset frequency range (e.g., <10 kHz) due to technical noise sources such as acoustic noise and power supply noise in pump lasers. This large technical-noise-originated timing jitter often limits the achievable synchronization performances of Ti:sapphire mode-locked lasers. Thus, in order to design an optimized synchronization phase-locked loop (PLL), we measured the timing jitter power spectral density (PSD) of the Ti:sapphire photocathode laser used in this work (Coherent Vitara-T with 79.33-MHz repetition-rate). Figure 1 shows the measurement results. Curve **a** represents the measured timing jitter PSD of the Ti:sapphire laser. Note that the PSD for <10 kHz offset frequency is measured by a commercial signal source analyzer (SSA), whereas the PSD for >10 kHz offset frequency is measured by the balanced optical cross-correlator (BOC)-based method^17^ for higher measurement resolution (see Methods for more information). For comparison, we also show the timing jitter PSD of a typical stretched-pulse mode-locked Er-fibre laser^18^ (curve **b**). Despite similar jitter level in the >10-kHz offset frequency range with 2.9-fs integrated timing jitter, the timing jitter PSD of the Ti:sapphire laser is more than 20 dB higher than that of the typical Er-fibre laser in the <10-kHz offset frequency range. This measurement result suggests that, for high-quality laser-RF synchronization, effective suppression of large timing jitter in the 100 Hz–10 kHz range is required. This is difficult to achieve for many commercial Ti:sapphire lasers with a limited PZT bandwidth and tuning range. As will be shown later, we therefore employed a noise eater and an extra-cavity PZT controller with extended bandwidth to further suppress jitter in this acoustic frequency range.

### Laser–RF synchronization methods

**Figure 2:**
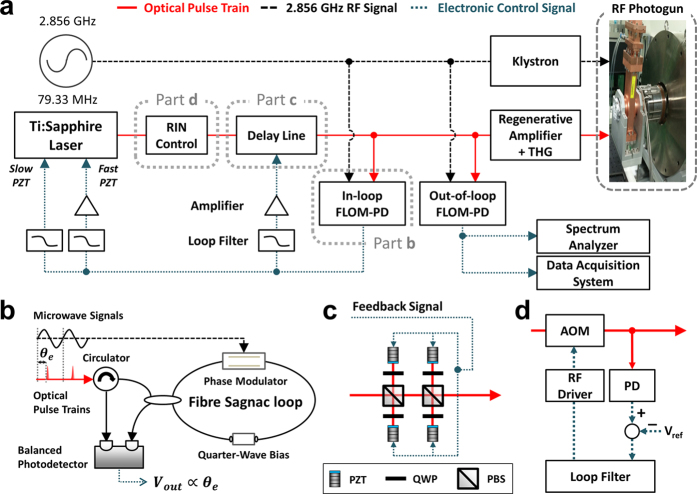
(**a**) Overall schematic of the laser-RF synchronization system. RIN, relative intensity noise. THG, third-harmonic generation. (**b**) Schematic of the fibre-loop optical-microwave phase detector (FLOM-PD). (**c**) Schematic of the extra-cavity delay control method. PBS, polarization beam splitter. QWP, quarter wave plate. (**d**) Schematic of extra-cavity RIN controller. AOM, acousto optic modulator. PD, photodiode.

Figure 2a shows the overall schematic of the laser-RF synchronization system. The central sub-system for precise laser-RF synchronization in this work is the optoelectronic PLL with an 2.856-GHz RF oscillator (Keysight N5181B) as the master oscillator, a 79.33-MHz Ti:sapphire photocathode laser oscillator as the slave oscillator, and an 800-nm fibre-loop optical-microwave phase detector (FLOM-PD)^2^ (Fig. 2b) as the phase detector.

By using a differential-biased fibre Sagnac-loop interferometer, the FLOM-PD enables timing detection between the optical pulse trains and the microwave zero-crossings with both sub-fs short-term resolution and few-fs long-term stability^2^. As a result, FLOM-PDs working at 1550-nm telecommunication wavelength have been widely used in photonic microwave/RF generation^19^, RF phase transfer over fibre links^20^,^21^, time synchronization fibre links^22^, and laser stabilisation^23^. More recently, a 800-nm-version FLOM-PD was demonstrated and successfully employed in a single-electron UED system for fs-precision timing diagnostics between a 6.2-GHz microwave signal and a 5-MHz Ti:sapphire laser^24^. In this work, 8 mW of input optical power and +18 dBm of input RF power to the FLOM-PD result in laser-RF phase detection sensitivity of 1.7 V/rad at 2.856 GHz (0.03 mV/fs). The detection background noise floor is −148 dBc/Hz, which corresponds to 3.1 fs (55.6-μrad at 2.856 GHz carrier) detection resolution for 1-MHz bandwidth. The phase error signal generated from the in-loop FLOM-PD is applied to two (slow and fast) PZT actuators in the Ti:sapphire laser, so that the Ti:sapphire laser is locked to the RF oscillator.

**Figure 3:**
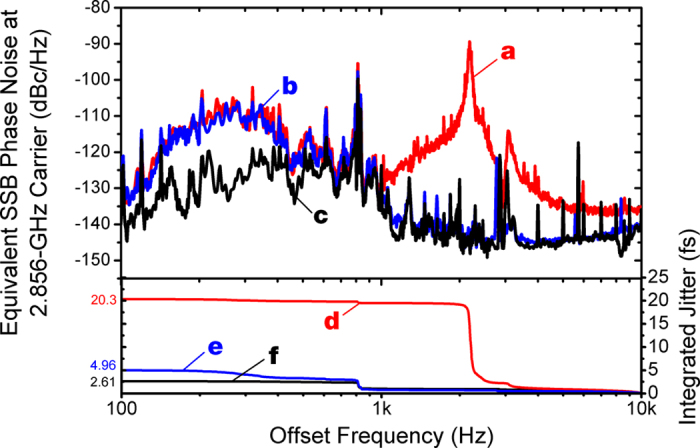
Residual phase noise (**a**) without noise eater and extra-cavity delay control; (**b**) with noise eater but without extra-cavity delay control; (**c**) with noise eater and extra-cavity delay control. Curves (**d**,**e** and **f)** are integrated timing jitter for curves (**a**,**b** and **c**), respectively.

As is shown in Fig. 1, technical noise in the Ti:sapphire lasers in the 100 Hz–10 kHz limits the achievable synchronization performance. To address this problem, we employed two additional methods. First, as shown in ref. 25, we inserted a noise eater circuit in the control cable to the Ti:sapphire laser. As shown in curves a and b of Fig. 3, the noise eater could effectively suppress the timing jitter in the 1 kHz–10 kHz range. Second, we implemented an extra-cavity delay control (Fig. 2c) for more extended actuator range and broader PLL bandwidth. To achieve both large tuning range and large bandwidth, four PZT-mounted mirrors are used. As a result, using a PZT with 8.8-μm displacement, we could extend the total displacement range up to 8.8 μm × 8 = 70.4 μm with 8.5-kHz resonant frequency. This displacement is equivalent to >230 fs peak-to-peak delay, which is sufficient to suppress the timing jitter in the 100 Hz–1 kHz range as shown by curve c of Fig. 3. By employing the external-cavity delay control and noise eater, the residual phase noise in the acoustic frequency range is significantly suppressed, which enables the residual rms jitter reduction from 20.3 fs to 2.9 fs.

Finally, to mitigate the amplitude-to-phase (AM-to-PM) conversion in FLOM-PD^26^ and also to enhance the laser power stability of the overall system, we added an extra-cavity RIN controller (Fig. 2d) using an acousto-optic modulator (AOM). By using the RIN controller, the laser output stability is improved from 0.18%rms fluctuation to 0.11%rms over 10,000 s, when measured with 2 samples/s and 1-Hz low-pass filter bandwidth.

To evaluate the overall laser-RF synchronization performance in the out-of-loop manner, we implemented an out-of-loop FLOM-PD and measured the residual timing jitter PSD and the timing drift by using spectrum analysers and data acquisition systems (see Methods for more information).

### Measurement results

**Figure 4:**
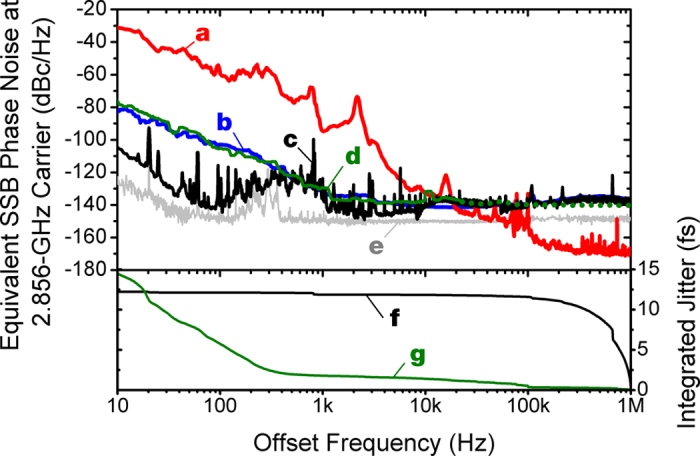
Phase noise measurement results. (**a**) Absolute phase noise of the Ti:sapphire laser (scaled to 2.856-GHz). (**b**) Absolute phase noise of the RF oscillator. (**c**) Residual phase noise between the laser and the RF oscillator measured by the out-of-loop FLOM-PD. (**d**) Absolute phase noise of the locked Ti:sapphire laser (measured by photodiode and signal source analyser). Note that the data for >20 kHz is limited by the measurement noise floor. (**e**) Background noise floor of the FLOM-PD. (**f**) Integrated residual timing jitter of synchronization (integration of curve (**c**)). (**g**) Integrated absolute timing jitter of RF-locked optical pulse train (combining curve (**d**) in the 10 Hz–20 kHz and curve (**a**) in the 20 kHz–1 MHz).

Figure 4 summarizes the short-term phase noise measurement results. Curves **a** and **b** are the absolute phase noise (scaled to 2.856 GHz carrier frequency) of the Ti:sapphire laser and the RF oscillator, respectively. By locking the Ti:sapphire laser to the RF oscillator with ~20 kHz PLL bandwidth, we can achieve the best absolute phase noise in optical pulse trains by combining the phase noise of the RF oscillator (inside the locking bandwidth) and the mode-locked laser (outside of the locking bandwidth). Curve **c** shows the measured laser-RF synchronization performance, i.e., the residual phase noise between the laser and the RF oscillator, measured by the out-of-loop FLOM-PD. The integrated residual rms timing jitter is 3.9 fs (12.2 fs) when integrated from 10 Hz to 100 kHz (1 MHz) offset frequency. In terms of residual phase error at 2.856 GHz carrier frequency, the performance corresponds to 0.070 mrad (0.22 mrad) over 100 kHz (1 MHz) bandwidth. Note that the rms timing jitter is 14.1 fs when integrating from 1 Hz to 1 MHz Fourier frequency. When measured by the FLOM-PD, the phase noise outside the locking bandwidth follows the absolute phase noise of RF oscillator, and the integrated residual jitter is mostly limited by the high-frequency (>100 kHz) phase noise of the RF oscillator itself (see curve **f**). Provided a better RF source with lower phase noise in the high offset frequency, the synchronization performance can be improved as well.

Note that, by the PLL flywheel effect, the absolute timing jitter of optical pulse train is not limited by the phase noise of the RF oscillators and follows the free-running laser jitter outside the locking bandwidth. Curve **d** is the absolute phase noise spectrum of the RF oscillator-locked Ti:sapphire laser, measured by direct photodetection and signal source analyser. As expected, inside the locking bandwidth (~18 kHz), the absolute phase noise of the Ti:sapphire laser follows that of the RF oscillator (curve **b**). Note that, however, due to the limited measurement resolution of direct photodetection method at ~−140 dBc/Hz, we could not properly measure the phase noise spectrum for >20 kHz Fourier frequency. As the measured resonant peak around PLL locking bandwidth is very weak in curve **d**, the absolute rms timing jitter of the locked optical pulse train can be properly estimated by combining the absolute phase noise spectra of the locked (curve **d**) and free-running (curve **a**) Ti:sapphire lasers in the 10 Hz–20 kHz and 20 kHz–1 MHz offset frequency ranges, respectively. As a result, the absolute jitter of the optical pulse train is reduced from 6.14 ps to ~14.5 fs when integrated from 10 Hz to 1 MHz (curve **g**).

**Figure 5:**
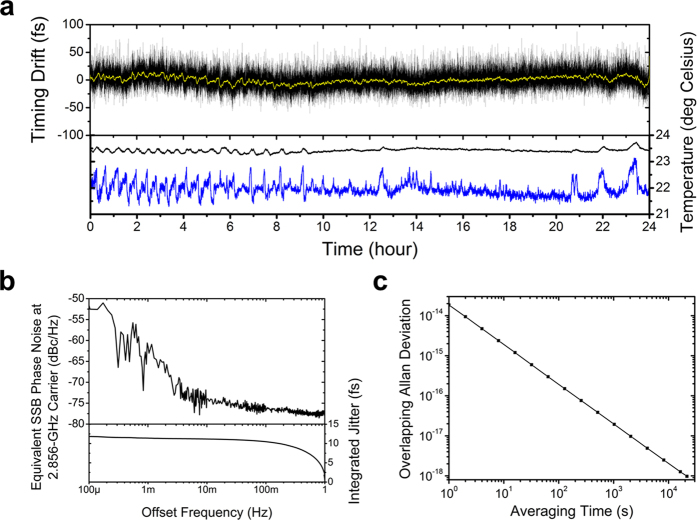
Long-term timing and phase drift measurement results. (**a**) Timing drift measurement data over 24 hours with 1-Hz bandwidth (black) and 0.01-Hz bandwidth (yellow). (**b**) Residual phase noise PSD from 0.1 mHz to 1 Hz. (**c**) Relative frequency instability in terms of overlapping Allan deviation.

Figure 5 shows the long-term timing stability measurement results. The output from the out-of-loop FLOM-PD is low-pass-filtered with 1-Hz bandwidth and sampled at 2 samples/s. The measured rms timing drift is maintained at 12.5 fs (0.22 mrad at 2.856 GHz) over 24 hours (Fig. 5a), which corresponds to the relative frequency instability of 9.8 × 10^−19^ at 21,600 s in terms of overlapping Allan deviation (Fig. 5c). When further taking low-pass filtering to the measured drift data with 0.01 Hz bandwidth (yellow curve in Fig. 5a), the slower drift corresponds to 5.8 fs (rms) over 24 hours. This is also confirmed by the phase noise PSD (Fig. 5b) computed from the drift data, which shows that vast majority of the drift is concentrated in the 0.01–1 Hz offset frequency range. Despite the extra-cavity RIN control, RIN in this frequency range (0.01–1 Hz) is not well suppressed, and the amplitude-to-phase conversion in the FLOM-PD is the main reason for the timing drift in the 100-s time scale. Provided better RIN suppression, the drift can be further suppressed.

## Discussion

In this paper, we demonstrate modular methods that achieve long-term-stable 10-fs-level synchronization between a commercial Ti:sapphire photocathode laser and a 2.856-GHz RF oscillator. This method may find many applications in ultrafast electron and X-ray sources, since precise laser-RF synchronization is a common requirement for such facilities. Also note that the synchronization techniques and noise analysis methods shown in this paper can be directly applied to other types of mode-locked solid-state lasers as well, for example, photocathode lasers with different gain media (such as Nd:YAG, Nd:YLF, and Yb:YAG) or seed lasers for seeded XFELs.

Note that the demonstrated system is successfully installed and operating at the UED/THz-FEL Facility^27^,^28^ of the Center for Quantum-Beam-based Radiation Research, Korea Atomic Energy Research Institute (KAERI). Currently, additional timing jitter/drift measurement and control is under way in the KAERI Facility. The tasks include the measurement and control of timing jitter and drift in regenerative amplifier^29^,^30^ and in free-space transfer^31^,^32^ from the regenerative amplifier to the RF photogun. The final laser-electron timing metrology methods at the UED target by using a deflecting RF cavity^33^ and spectral decoding of electron-beam-generated THz pulses on chirped laser pulses^34^ are also under way.

As a final note, the timing jitter number can be sometimes misleading to properly assess the laser-RF synchronization performance. First, the jitter number is a function of integration bandwidth, and many previous works show jitter results with <100 kHz integration bandwidth^7^,^9^,^10^. Second, the jitter number inversely scales with the used RF frequency. For example, with the same residual phase noise performance in synchronization, the integrated jitter number will be reduced from 12.2 fs to 3.5 fs if the used RF frequency is increased from 2.856 GHz to 10 GHz. Therefore, the residual phase noise level and integration bandwidth should be carefully examined when comparing results from different systems.

## Methods

### Timing jitter measurement of the Ti:sapphire laser

To measure the absolute timing jitter of the used free-running Ti:sapphire laser in the low (<20 kHz) offset frequency, the laser output is detected with a 2-GHz Si p-i-n photodiode and bandpass-filtered at 1.43 GHz (18th harmonic of 79.33 MHz). The phase noise of the filtered signal is measured by a signal source analyser (Rodhe & Schwarz, FSUP26). To measure the jitter in the high (>10 kHz) offset frequency for free-running Ti:sapphire laser, we built a two-colour balanced optical cross-correlator^35^. The Ti:sapphire laser is locked in repetition-rate with a lower-jitter 1550-nm solid-state laser with 800 Hz locking bandwidth, and the jitter spectrum outside the locking bandwidth is analysed.

### 800-nm FLOM-PD implementation

The FLOM-PD is implemented by the standard procedures shown in refs 2 and 24 using single-mode polarization-maintaining fibre and fibre-coupled devices working at 800 nm. It is sealed by Derlin case to minimize the impact of laboratory temperature changes. To maintain the temperature inside the case, the FLOM-PD is built on the water-cooled breadboard.

### Residual phase noise, timing jitter and timing drift measurement methods

The output of the out-of-loop FLOM-PD is used to measure the residual phase noise, timing jitter and drift. An FFT spectrum analyser (Standford Research Systems, SR785) and an RF spectrum analyser (Keysight, E4411B) are used to measure the residual phase noise spectra in the 10 Hz–100 kHz and the 100 kHz–1 MHz offset frequency, respectively. For measuring residual timing drift, the output of the out-of-loop FLOM-PD is lowpass filtered at 1 Hz (Standford Research Systems, SR560) and sampled at 2 samples/s using the data acquisition board (National Instrument, USB-6211).

## Additional Information

**How to cite this article**: Yang, H. *et al*. 10-fs-level synchronization of photocathode laser with RF-oscillator for ultrafast electron and X-ray sources. *Sci. Rep.* **7**, 39966; doi: 10.1038/srep39966 (2017).

**Publisher's note:** Springer Nature remains neutral with regard to jurisdictional claims in published maps and institutional affiliations.
